# Endovascular treatment of thoracic aortic pseudoaneurysm due to brucellosis: a rare case report

**DOI:** 10.1186/s12879-017-2485-7

**Published:** 2017-06-02

**Authors:** Shuai Wang, Qi Wang, Han Liu, Siqiao Sun, Xiwei Sun, Yang Zhang, Zhongying Wang, Zhihua Cheng

**Affiliations:** 1 Department of Vascular Surgery, the First Hospital of Jilin University, Xinmin Street 71, Changchun, Jilin, China; 2 Department of Respiration, the First Hospital of Jilin University, Xinmin Street 71, Changchun, Jilin, China

**Keywords:** Brucellosis, Pseudoaneurysm, Thoracic aorta, Stent implantation

## Abstract

**Background:**

Arterial damage is a known complication of brucellosis, but the occurrence of a thoracic aortic pseudoaneurysm secondary to brucellosis has not been previously reported.

**Case presentation:**

A 65-year-old Chinese man presented with a pseudoaneurysm in the descending segment of the thoracic aorta that caused symptoms of chest pain and intermittent fever. He was diagnosed with a thoracic aortic pseudoaneurysm secondary to brucellosis based on a positive *brucella* serology test (standard-tube agglutination test) and imaging examination (computed tomography angiography). Anti-brucellosis treatment and covered stent graft implantation were attempted to eliminate the brucellosis and pseudoaneurysm, respectively, and were ultimately successful, with no symptoms after 6 months of follow-up.

**Conclusion:**

Endovascular repair may be effective and safe for treating a thoracic aortic pseudoaneurysm resulting from brucellosis.

## Background

Brucellosis is a zoonotic infection that most commonly occurs in Middle Eastern, Mediterranean, and East Asian countries. Nearly 200 cases are reported every year in Italy, [1] and nearly 4000 cases are reported every year in China, with more than half of the cases occurring in the northeast and northern regions, including the inner Mongolia autonomous region and Heilongjiang and Jilin provinces. The reservoir hosts include sheep, cattle, and swine, and transmission from sheep is the most common way of contraction in China [2]. Humans become infected with the disease through ingestion of contaminated milk products or meat or through direct contact via the mucous membranes. Certain professions, such as breeder, butcher, dairyman, and kitchen employee, are associated with an increased risk of contracting the disease [2]. The main clinical presentations include undulant fever, night sweats, and joint pain in the acute stage [3]. These symptoms as well as joint damage, tendon contracture, meningitis, orchitis, and epididymitis are all very common in the chronic stage. Meanwhile, damage to the cardiovascular, digestive, and respiratory systems is occasionally observed. More than 75% of deaths related to brucellosis occurred due to endocarditis [3, 4]. Pseudoaneurysms in peripheral arteries have been reported in a few cases, [5–7] but there has been no report of the occurrence of a thoracic aortic pseudoaneurysm secondary to brucellosis. Here, we describe a rare case of a brucellosis-induced thoracic aortic pseudoaneurysm and discuss the treatment procedure applied for this patient.

## Case presentation

**Fig. 1:**
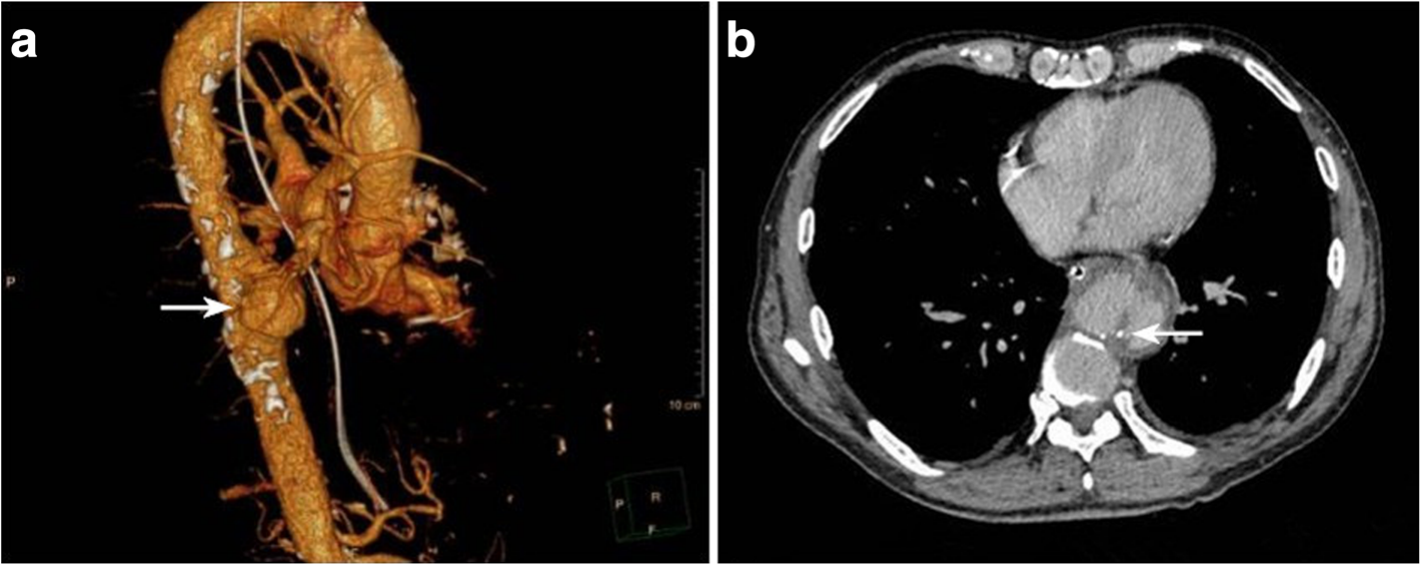
Computed tomography angiography (CTA): **a**. The pseudoaneurysm was ound in the descending segment of the thoracic aorta, with a maximal diameter of 54 mm (*arrow*). **b**. Arteriosclerotic plaques were observed in the artery wall (*arrow*)

**Fig. 2:**
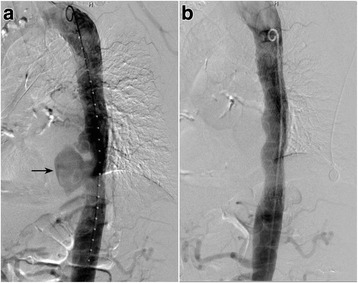
Digital subtraction angiography (DSA): **a**. The pseudoaneurysm was revealed by contrast medium overflow (*arrow*). **b**. Angiography showed no contrast medium overflow in the thoracic aorta after treatment

A 65-year-old Chinese man was admitted to the First Hospital of Jilin University complaining of intermittent fever for 2 months with the highest temperature reaching 40 °C and chest pain for 10 days. The patient had no history of drug abuse, invasive examination, thoracic aortic surgery, or trauma. Upon physical examination, the patient presented with low-grade fever with normal blood pressure, heart rate, and heart rhythm. Additional imaging examination via computed tomography angiography (CTA, **(**Fig. 1 **)** revealed the formation of a pseudoaneurysm with a maximal diameter of 54 mm in the descending segment of the thoracic aorta. Electrocardiogram (ECG) showed normal sinus rhythm. Transthoracic echocardiography (TTE) was performed and showed mild mitral insufficiency without valvular vegetation. On additional laboratory tests, the C-reactive protein (CRP) level was 106 mg/L, the erythrocyte sedimentation rate (ESR) was 97 mm/h, the white blood cell count (WBC) was 7.6 × 10^12^/L with 83% neutrophils, and the hemoglobin (Hb) level was 106 g/L. The results of neurological and cardiovascular examinations were normal, and the patient had no significant medical history of hypertension, heart diseases, diabetes mellitus, blood transfusion, genital ulcer, and/or skin rash. Therefore, it seemed unlikely that the case was caused by complications of these diseases. However, the patient recounted that he had been employed in a family owned restaurant and exposed to cattle and raw meat for 2 years since he had retired from being a sailor 4 years previously. Considering this information, a blood bacterial culture was ordered. A definitive diagnosis was made by isolating the *brucella* in the blood culture, and the finding on the standard-tube agglutination test (SAT) was 1:400, which corresponded to a strong positive result. In view of an active brucellosis infection and the high vascular inflammatory reaction that can cause a series of complications if surgery or endovascular treatment were to be performed, a combination of anti-brucellosis treatment with 450 mg rifampin twice a day orally and 200 mg doxycycline once a day orally was started immediately. We advised the patient to continue the treatment for at least 6 months. However, the patient presented with aggravated chest pain and mild hemoptysis (bright red, nearly 10 ml) without obvious cause 2 days later, which were considered the result of pseudoaneurysm rupture and hemorrhage into the pulmonary alveoli. To minimize the risks of morbidity and mortality in the repair of the thoracic aortic pseudoaneurysm, the Tag covered stent graft (W. L. Gore & Associates, USA) was implanted to isolate the pseudoaneurysm. Intraoperatively, a 15-cm, 6F sheath (Terumo Corporation, Japan) was advanced into the right common femoral artery in a retrograde fashion. The pseudoaneurysm was viewed on digital subtraction angiography (DSA) by a pigtail catheter **(**Fig. 2 **).** Then a 28-cm, 22F sheath (W. L. Gore & Associates, USA) was advanced, and a 31 mm × 150 mm Tag covered stent graft (W. L. Gore & Associates, USA) was implanted to isolate the pseudoaneurysm through a Lunderquist extra stiff wire guide (Cook Medical Inc., USA). Finally, the angiography showed no contrast medium overflow in the thoracic aorta **(**Fig. 2 **).** Postoperatively, the anti-brucellosis treatment was continued, and the symptoms disappeared gradually over the first week. At the 24th week follow-up, the SAT result was 1:50, which corresponded to a negative result. The patient continued the anti-brucellosis treatment for 2 more weeks, and at the 26th week follow-up, the SAT was negative and the patient was well and asymptomatic.

## Discussion

*Brucella* can infect endothelial cells and induce a severe inflammatory response, leading to possible cardiovascular complications. In the inflammatory response, the pathogenic mechanism is suggested to involve endothelial activation in response to *brucella* infection, with upregulation of adhesion molecules and secretion of proinflammatory chemokines [8]. Vascular complications of brucellosis are rare, but include aneurysm in the peripheral arteries (aorta, brachial, tibioperoneal, mesenteric, and cerebral arteries) and venous and arterial thrombosis with or without underlying endocarditis [9–15]. Although rare, such vascular complications are so deadly they are the primary cause of death related to brucellosis. Pseudoaneurysm secondary to brucellosis is extremely rare in the peripheral arteries, but it is a very severe and dangerous complication [5–7]. In other case studies, it has been reported that mycotic pseudoaneurysm usually is due to trauma to the arterial wall with subsequent contamination [6, 16–21]. The arterial segment might be disrupted when an invasive infection is able to directly lodge in the artery or the vasa vasorum, especially with an intimal defect in the arterial wall such as an arteriosclerotic plaque [6, 19, 20]. In our case, the arteriosclerotic plaques were on CTA imaging.**(**Fig. 1 **)** Due to the arteriosclerotic plaques and damage to the arterial endothelium, the medial layer was exposed to the bloodstream. Based on the physiological and pathological processes, *brucella* can easily destroy and even penetrate the arterial wall.

Generally, most cases of thoracic aortic pseudoaneurysms in the hospital settings occur following thoracic aortic surgery or trauma and are associated with high mortality rates [22]. Thus, urgent treatment of the thoracic aorta pseudoaneurysm is needed. Compared with open surgery, the endovascular treatment is more likely to minimize the operative wound, hospital stay, and peri-operative mortality [23]. In view of these factors, we performed the minimally invasive operation of implanting a coated stent graft and achieved an ideal outcome in our case. Management of *Brucella*-induced pseudoaneurysm involves resection of the affected artery and endovascular stenting together with long-term anti-brucellosis treatment. Despite the known hepatotoxicity, neurotoxicity, and reproductive toxicity, rifampin, doxycycline, and streptomycin are the basic drugs used for anti-brucellosis treatment clinically. Without a normative anti-brucellosis treatment, the patient would be more likely to suffer a poor prognosis [24].

## Conclusions

In conclusion, the present case may be the first reported case of *Brucella*-induced thoracic aortic pseudoaneurysm and was successfully managed by endovascular treatment. However, this case has some limitations: 1. the follow-up time was ony 6.5 months; 2. the *Brucella*-infected arterial wall and tissue were not removed; and 3. the diagnosis was based on a serological test (SAT) and imaging examination (CTA) without pathological analysis. Thus, a case series study is needed to facilitate a systematic diagnosis and formulate treatment recommendations for thoracic aortic pseudoaneurysm caused by brucellosis.

## Abbreviations

CRP: C-reactive protein
CTA: Computed tomography angiography
DSA: Digital subtraction angiography
ECG: Electrocardiogram
ESR: Erythrocyte sedimentation rate
Hb: Hemoglobin
SAT: Standard-tube agglutination test
TTE: Transthoracic echocardiogram
WBC: White blood cell count
